# The genetic basis of chloride exclusion in grapevines

**DOI:** 10.1093/g3journal/jkaf149

**Published:** 2025-06-30

**Authors:** Sadikshya Sharma, Noe Cochetel, Jose R Munoz, Hollywood Banayad, Yaniv Lupo, Veronica Nunez, Ana Gaspar, Christopher Chen, Krishna Bhattarai, Brandon S Gaut, Dario Cantu, Luis Diaz-Garcia

**Affiliations:** Department of Viticulture and Enology, University of California, Davis, Davis, CA 95616, United States; Department of Viticulture and Enology, University of California, Davis, Davis, CA 95616, United States; Department of Viticulture and Enology, University of California, Davis, Davis, CA 95616, United States; Department of Viticulture and Enology, University of California, Davis, Davis, CA 95616, United States; Department of Viticulture and Enology, University of California, Davis, Davis, CA 95616, United States; Department of Viticulture and Enology, University of California, Davis, Davis, CA 95616, United States; Department of Viticulture and Enology, University of California, Davis, Davis, CA 95616, United States; Cooperative Extension, Division of Agriculture and Natural Resources, Hopland Research and Extension Center, University of California, Hopland, CA 95449, United States; Department of Horticultural Sciences, Texas A&M AgriLife Research and Extension Center, Texas A&M University, Dallas, TX 75252, United States; Department of Ecology and Evolutionary Biology, University of California, Irvine, Irvine, CA 92697, United States; Department of Viticulture and Enology, University of California, Davis, Davis, CA 95616, United States; Department of Viticulture and Enology, University of California, Davis, Davis, CA 95616, United States

**Keywords:** salinity tolerance, grapevine rootstocks, Vitis diversity, rootstock breeding, Plant Genetics and Genomics

## Abstract

Mediterranean regions are among the most important areas for global grape production, characterized by dry climates and frequent challenges associated with soil salinity. In these environments, chloride toxicity is a major factor limiting vine growth and fruit quality. Despite the critical role of chloride exclusion in salinity tolerance, the genetic mechanisms underlying this trait remain poorly understood. In this study, we analyzed natural variation in chloride exclusion using a diverse panel of 335 accessions representing 18 wild and cultivated *Vitis* species. This panel, comprising accessions from the southwestern United States and Mexico, captures a broad range of evolutionary adaptations to abiotic stress and provides a valuable genetic resource for breeding efforts aimed at introducing novel traits. Using genome-wide association and quantitative trait loci (QTL) mapping, we identified a major QTL on chromosome 8, now designated qClEx8.1, containing candidate genes encoding cation/H⁺ exchangers (CHXs), which are involved in ion transport and homeostasis. To validate these findings, we analyzed a mapping population derived from *Vitis acerifolia* longii 9018 and the commercial rootstock GRN3, confirming the chromosome 8 locus as a major determinant of chloride exclusion. Structural variant analysis revealed nonsynonymous substitutions within CHX genes that may influence protein function and salinity tolerance. Additionally, we discovered a novel QTL on chromosome 19 enriched with G-type lectin S-receptor-like serine/threonine-protein kinases, known regulators of stress signaling. By integrating phenotypic and genomic data across a diverse *Vitis* collection, this study advances our understanding of the genetic architecture underlying chloride exclusion and highlights candidate genes for breeding salt-tolerant rootstocks.

## Introduction

Soil salinization is a major abiotic stress affecting agriculture worldwide. Approximately 1.4 billion hectares of land worldwide are affected by salinity, with an additional 1 billion hectares at risk (FAO 2024). The primary contributors to soil salinization include inadequate irrigation practices, poor-quality irrigation water, overexploitation of water resources, and climate change-induced factors such as rising temperatures and altered precipitation patterns (Olesen et al. 2011; Ollat *et al.* 2016). Grape production is particularly relevant in semi-arid or Mediterranean-type climates, where limited rainfall and higher evaporation rates can exacerbate salt accumulation, making vines particularly vulnerable. Soil salinity not only reduces grape yield but also impacts fruit quality (Walker *et al.* 2003). Excessive sodium (Na^+^) and chloride (Cl^−^) accumulation in plant tissues can induce ion toxicity symptoms such as leaf necrosis and reduced photosynthesis, leading to stunted vegetative growth, leaf burn, and defoliation (Walker *et al.* 2010; Baby *et al.* 2016). Studies have shown that grapevines are moderately sensitive to salinity, with growth reductions starting at soil electrical conductivity (ECe) levels of 1.5 dS/m, and yield losses of ∼9.6% for each additional dS/m increase (Grieve *et al.* 2012). Beyond yield reductions, high soil salinity disrupts the balance of sugars, acids, and flavor compounds in grapes, ultimately affecting the resulting wine's enological properties and quality (Walker *et al.* 2003). As climate change intensifies, the frequencies of hot and dry periods are expected to rise, exacerbating soil salinization and further threatening grape production (Walker *et al.* 2003; Munns and Tester 2008; Hannah *et al.* 2013; Baby *et al.* 2016 ; Phogat *et al.* 2020).

Grapevines employ multiple strategies to tolerate salt stress, including shoot ion exclusion (Henderson *et al.* 2014; Heinitz *et al.* 2020), vacuolar sequestration of intracellular ions (Wu *et al.* 2019), reactive oxygen species (ROS) signaling and detoxification (Zhang *et al.* 2016; Tanveer and Ahmed 2020), and osmotic adjustment through accumulation of organic osmolytes (Sharma *et al.* 2019; Van Zelm *et al.* 2020). Salt exclusion, a key mechanism, enables plants to limit the uptake of Na^+^ and Cl^−^ from the soil and/or restrict their translocation from roots to shoots via the xylem (Wu *et al.* 2019). While the genetic and physiological mechanisms governing Na^+^ exclusion are relatively well understood (Mohammadkhani *et al.* 2016; Henderson *et al.* 2018; Wu *et al.* 2020; da Silva *et al.* 2024), Cl^−^ exclusion remains less characterized. Despite both Na^+^ and Cl^−^ contributing to salinity stress, grapevine rootstocks have traditionally been classified as tolerant or susceptible based on their ability to restrict Cl^−^ uptake, with Na^+^ exclusion considered a secondary factor (Walker 1994; Walker *et al.* 2003; Heinitz *et al.* 2020). This distinction is particularly significant in California, one of the world's largest grape-producing regions, where Cl^−^ severely impacts many major viticultural areas, making chloride exclusion a key trait for the development of salt-tolerant rootstocks (Fort *et al.* 2013, 2015; Heinitz *et al.* 2020; Sharma *et al.* 2024).

Wild *Vitis* species exhibit greater variability in Cl^−^ exclusion than most commercially available rootstocks, highlighting their potential as a genetic resource for improving salt tolerance. A comprehensive survey of 325 accessions from 14 *Vitis* species collected across the southwestern United States and Mexico revealed substantial variation in Cl^−^ exclusion, both between and within species (Heinitz *et al.* 2020). Some accessions exhibited strong chloride exclusion—accumulating significantly less Cl⁻ than 140 Ruggeri, a widely used commercial rootstock for high-salinity conditions (Sharma *et al.* 2024)—while others showed weak exclusion ability, underscoring considerable intraspecific variability. Certain species, such as *Vitis acerifolia*, consistently restricted Cl^−^ uptake, while others displayed a broad range of chloride accumulation, suggesting that multiple genetic mechanisms regulate this trait (Heinitz *et al.* 2020; Sharma *et al.* 2024). Building on these findings, Cochetel *et al.* (2023) identified a genomic region on chromosome 8 (position 13,598,495 bp) associated with chloride exclusion, based on the Heinitz *et al.* (2020) phenotypic survey. They discovered a predicted protein homologous to the *Arabidopsis thaliana* cation/H⁺ exchanger (CHX) AtCHX20 (*AT3G53720*) within this region. The putative function of this gene was further supported by the presence of 2 key InterPro domains: the CHX (IPR006153) and the sodium/solute symporter superfamily (IPR038770). Moreover, the gene upstream of the significant SNP was also found to be homologous to *AtCHX20*. To date, no other grapevine candidate genes, major or minor, have been identified for Cl^−^ exclusion.

In this study, we explored the extensive genetic diversity of 335 accessions spanning 18 *Vitis* species and uncovered remarkable variation in Cl⁻ exclusion both within and between species. This broad survey underscores the rich genetic potential within wild and cultivated *Vitis* germplasm for enhancing salt tolerance. Building on these findings, our genome-wide association study (GWAS) and linkage mapping not only confirmed the previously reported quantitative trait loci (QTL) on chromosome 8 (Cochetel *et al.* 2023)—a region containing CHX genes—but also identified an orthologous locus in *V. acerifolia* that reinforces the importance of these candidate genes. In addition, we discovered a novel QTL on chromosome 19 enriched with *G*-type lectin S-receptor-like serine/threonine-protein kinase genes known to play significant roles in salt stress adaptation. Collectively, these results advance our understanding of the genetic underpinnings of chloride exclusion, highlighting the power of exploiting natural *Vitis* diversity to develop improved, salt-tolerant rootstocks and cultivars.

## Materials and methods

### Plant material

This study utilized 335 Vitis accessions from the University of California, Davis, grapevine germplasm collection, which are clonally preserved on the UC Davis campus in Davis, CA, USA. The accessions represent 18 *Vitis* species, primarily from the southwestern United States, including *V. acerifolia*, *Vitis aestivalis*, *Vitis arizonica*, *Vitis berlandieri, Vitis californica*, *Vitis candicans*, *Vitis champinii*, *Vitis cinerea*, *Vitis × doaniana*, *Vitis girdiana*, *Vitis monticola*, *Vitis riparia*, *Vitis rupestris*, *Vitis treleasei*, and *Vitis vulpina*. This germplasm collection has been previously characterized for traits, such as disease resistance (Morales-Cruz *et al.* 2023), abiotic stress tolerance (Morales-Cruz *et al.* 2021), and genome composition (Cochetel *et al.* 2023). At the genomic level, various efforts have contributed to its characterization, including the development of reference genomes (Minio *et al.* 2022; Cochetel *et al.* 2023), whole-genome resequencing (Morales-Cruz *et al.* 2021, 2023), and the construction of a multi-species pangenome (Cochetel *et al.* 2023). Although several of these accessions, specifically 145 out of the 335, were previously evaluated for chloride exclusion in Heinitz *et al.* (2020) and subsequently included in a GWAS by Cochetel *et al.* (2023), all phenotypic data used in the present study are newly generated. No previously published phenotypes were reused. This approach ensured data consistency and comparability across the full set of accessions, especially considering the known variability in phenotypic expression across trials. A list of overlapping accessions between the present study and Heinitz *et al.* (2020) is provided in Supplementary Table 1, while passport data and chloride content for all accessions are provided in Supplementary Table 2.

### Experimental design

We conducted 4 independent greenhouse trials over 2 yr, evaluating between 61 and 141 accessions and 4 replicates per accession, over 2 yr to assess 335 *Vitis* accessions spanning 18 species. In total, 1,340 vines were examined. Green cuttings from the 335 accessions were collected during the spring over 2 growing seasons (2023 and 2024). Collections took place in the early morning (6:00 to 10:00 AM) to ensure tissue viability. Cuttings were treated with 2% indole-3-butyric acid before being placed in trays filled with presoaked perlite. The trays were maintained in a fog room (100% relative humidity and 27 °C) for 14 d to promote root development and bud emergence. Once rooted, the cuttings were transplanted into 4-inch pots containing fritted clay and grown for 2 mo before initiating the chloride stress treatment. Previous studies have established 50 mM NaCl as an appropriate concentration to induce salinity stress in potted grapevines using a similar screening method (Heinitz *et al.* 2020; Sharma *et al.* 2024). To ensure consistent salt exposure, each pot received 1 L of saltwater solution daily between 7:00 and 8:00 AM. Given the large number of accessions and replication levels, chloride-exclusion screening was conducted across 4 independent trials using an augmented design. Four accessions—140 Ru, longii 9018, St George, and 44 to 53 M—were included as controls in all trials, as they have been previously characterized as either chloride excluders or nonexcluders. The number of accessions and key dates for each trial are summarized in Supplementary Table 3.

Additionally, we generated a mapping population of 162 individuals by crossing the chloride-excluding accession *V. acerifolia* longii 9018 with the commercial rootstock GRN3. This population, designated 18113, was planted in the field, and a chloride-exclusion screening, similar to the one conducted with the GWAS population, was performed.

### Leaf chloride content phenotyping

After 21 d of salt treatment, all leaves and petioles from each plant were harvested and stored in a paper bag. The samples were air-dried in a drying room at 50 °C for 2 wk, then ground into fine powder. Subsequently, 0.25 g of the fine powder was mixed with 25 mL of deionized water in screw-cap bottles, following the protocol of Heinitz *et al.* (2020). The mixture in screw-cap bottles was shaken at 150 revolutions per minute for 1 h, then filtered through an 11-µm filter to obtain a clear solution without leaf particles. The chloride content of the filtered samples was then measured using a silver ion titration chloridometer (Model 926, Nelson-Jameson Inc.) following the manufacturer's calibration protocol with known chloride standards. Each sample was measured 3 times, and the average chloride concentration was expressed in mg/L.

The chloride content was analyzed using linear mixed models implemented in the *lme* package in R (Bates *et al.* 2015). We generated Best Linear Unbiased Predictors (BLUP) for each accession using the following model:


$$y_{ijk}=\mu +L_{i}+b_{k}+g_{ij}+c_{ij}+e_{ij},$$

where *y_ijk_* represents the phenotype of response variable *i* in trial *j* and block *k*; *μ* is the overall mean; *L_i_* is the fixed effect of trial; *b_k_* is the random effect of block *k* within each trail; *g_ij_* is the random genetic effect of regular individuals in trial *j*; *c_ij_* is the random genetic effect of experimental checks in trial *j*; and *e_ij_* is the random residual effect.

### Phylogenetic and population structure analysis

A phylogenetic analysis was performed to infer evolutionary relationships among accessions. A neighbor-joining tree using all the SNPs was constructed based on genetic distance allowing visualization of genetic divergence and the hierarchical relationships between the accessions using the R packages *ape* (Paradis *et al.* 2004) and *ggtree* (Yu *et al.* 2017). Population structure analysis was conducted using the sparse non-negative matrix factorization algorithm implemented in the *LEA* package in R (Frichot and François 2015). The optimal number of genetic clusters (*K*) was determined by evaluating cross-entropy values. The analysis was finalized with *K* = 9, and the admixture proportions (*Q*-matrix) were extracted based on the results.

### Genotyping and GWAS

Sequencing data for the 313 accessions used were compiled from previous work (Morales-Cruz *et al.* 2021, 2023; Cochetel *et al.* 2023) and are available at NCBI under BioProject IDs: PRJNA731597, PRJNA842753, and PRJNA984685. We filtered and processed raw sequencing reads using Trimmomatic-0.36 (Bolger *et al.* 2014) and FastQC (Andrews 2010). Reads were scanned in sliding windows of 4 base pairs, and bases were trimmed when the average quality per base dropped below Q20. We removed leading and trailing bases with quality scores below Q3 and retained only reads that were ≥60 bp after trimming. Filtered reads were then mapped to haplotype 1 of *V. girdiana* SC2 using the BWA-MEM algorithm implemented in bwa0.7.12-r1039 (Li 2013). Joint genotyping was performed using GATK v.4.0.12.0 (Van der Auwera and O'Connor 2020). We first marked duplicate reads using the “MarkDuplicates” function from Picard tools, followed by the “AddOrReplaceReadGroups” function to label reads by individual. For SNP prediction, we applied the HaplotypeCaller algorithm with a ploidy of 2 and a mapping base quality score threshold of Q20. The final SNP calls were generated by combining VCF files from all individuals using the “GenotypeGVCFs” function with default parameters. SNP filtering was conducted using bcftools v1.19 (Danecek *et al.* 2021), applying stringent criteria to retain high-confidence variants following GATK's guidelines for hard filtering. We kept only biallelic SNPs with, up to 25% missing data among individuals, and minor allele frequency (MAF) >0.1. Sites present in repetitive regions were discarded using bedtools v.2.31.1 (Quinlan and Hall 2010).

Genome-wide association was conducted using a mixed linear model implemented in the gemma v.0.98.3 (Zhou and Stephens 2012). The genomic relationship matrix (⁠⁠*K*) and 10 PCs, derived from SNP marker genotypes, were used to adjust for genetic relationships among individual. Prior to GWAS, PLINK v1.9 (Chang *et al.* 2015) was used for SNP pruning to reduce linkage disequilibrium (LD) among markers using “–indep-pairwise 50 5 0.2.” The pruned dataset was used to generate the standardized relatedness matrix to account for kinship among individuals. The significance threshold was established using false discovery rate (FDR) correction to identify SNPs that exceeded the genome-wide significance level. To pinpoint potential candidate genes within each QTL region, we leveraged positional gene information and functional annotations from our dataset (www.grapegenomics.com). Candidate genes were identified within a ±100 kb window surrounding each QTL.

### QTL mapping in the longii 9018 × GRN population

Genomic DNA was extracted from young leaf samples at the University of Minnesota Genome Center, while genotyping-by-sequencing (GBS) was performed at the University of California, Davis Genome Center using the Illumina NovaSeq 6000 platform with paired-end 150 bp reads and an average sequencing depth of 10× coverage. Variant calling was conducted using TASSEL (Glaubitz *et al.* 2014), with haplotype 1 of *V. acerifolia* longii 9018 as the reference genome. To ensure high-quality variant calls, reads with a depth below 20 or above 200 were marked as missing, along with heterozygous calls exhibiting skewed allele proportions. Markers with >25% missing data and individuals with >40% missing genotype information were excluded from further analysis. Additionally, distorted markers were removed based on a χ^2^ test at *P* < 0.05.

Two genetic maps, 1 for longii 9018 and another for GRN3, were constructed using a pseudo-test cross linkage mapping strategy (Grattapaglia and Sederoff 1994) in BatchMap (Schiffthaler *et al.* 2017). To generate predictors for the progeny population, we applied a similar model to the 1 used for GWAS BLUP estimation. A single-marker regression approach was implemented in R/qtl (Broman *et al.* 2003), with genome-wide significance thresholds established through 1,000 permutation tests.

## Results

### Interspecies and intraspecies variation for chloride exclusion

**Fig. 1.**
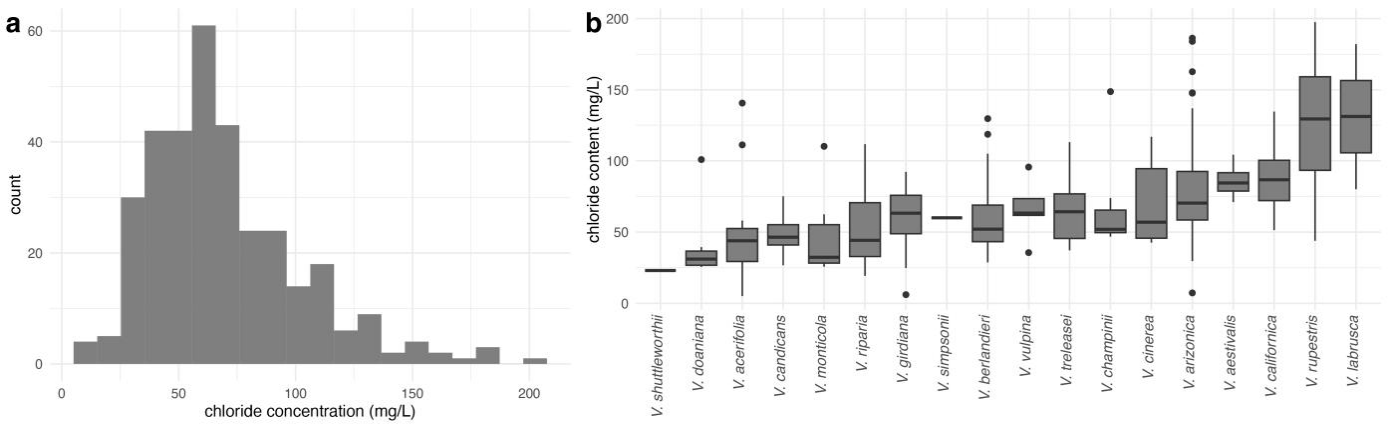
Variability in leaf Cl^−^ content across 335 accessions and 18 *Vitis* species. a) Histogram of the average leaf Cl^−^ content using BLUPs for all the accessions. b) Leaf Cl^−^ content across 18 *Vitis* species.

Chloride exclusion was measured as the amount of Cl^−^ in the leaves after 21 d of growth under 50 mM NaCl. The distribution of Cl⁻ exclusion was continuous and skewed toward lower chloride content (Fig. 1a), with high variability observed both between and within species. BLUPs ranged from 5.07 (OK14-006, *V. acerifolia*) to 197.45 mg/L (Vru 110, *V. rupestris*), with a mean of 69.45 mg/L. In addition, notable interspecific differences were observed (Fig. 1b). Among those with the lowest mean values, *Vitis shuttleworthii* exhibited the lowest chloride content (23.0 mg/L), although this estimate is based on a single sample. Similarly, *V. × doaniana* (mean = 39.6 mg/L, *n* = 8) and *V. acerifolia* (mean = 46.4 mg/L, *n* = 17) also displayed low mean chloride levels. In contrast, species with higher chloride contents included *Vitis labrusca* (mean = 131.0 mg/L, *n* = 2), *V. rupestris* (mean = 126.0 mg/L, *n* = 10), and *V. californica* (mean = 88.6 mg/L, *n* = 6). The largest intraspecific variation was found in *V. labrusca* (SD = 72.1, *n* = 2), *V. rupestris* (SD = 48.8, *n* = 10), and *V. champinii* (SD = 36.7, *n* = 17), whereas *V. candicans* (SD = 11.8, *n* = 25), *V. aestivalis* (SD = 13.9, *n* = 4), and *V. vulpina* (SD = 21.7, *n* = 5) had the lowest. The number of accessions sampled per species did not correlate with the standard deviation (SD; *r* = 0.05, *P* = 0.8333).

### Phylogenetic and population structure analysis

**Fig. 2.**
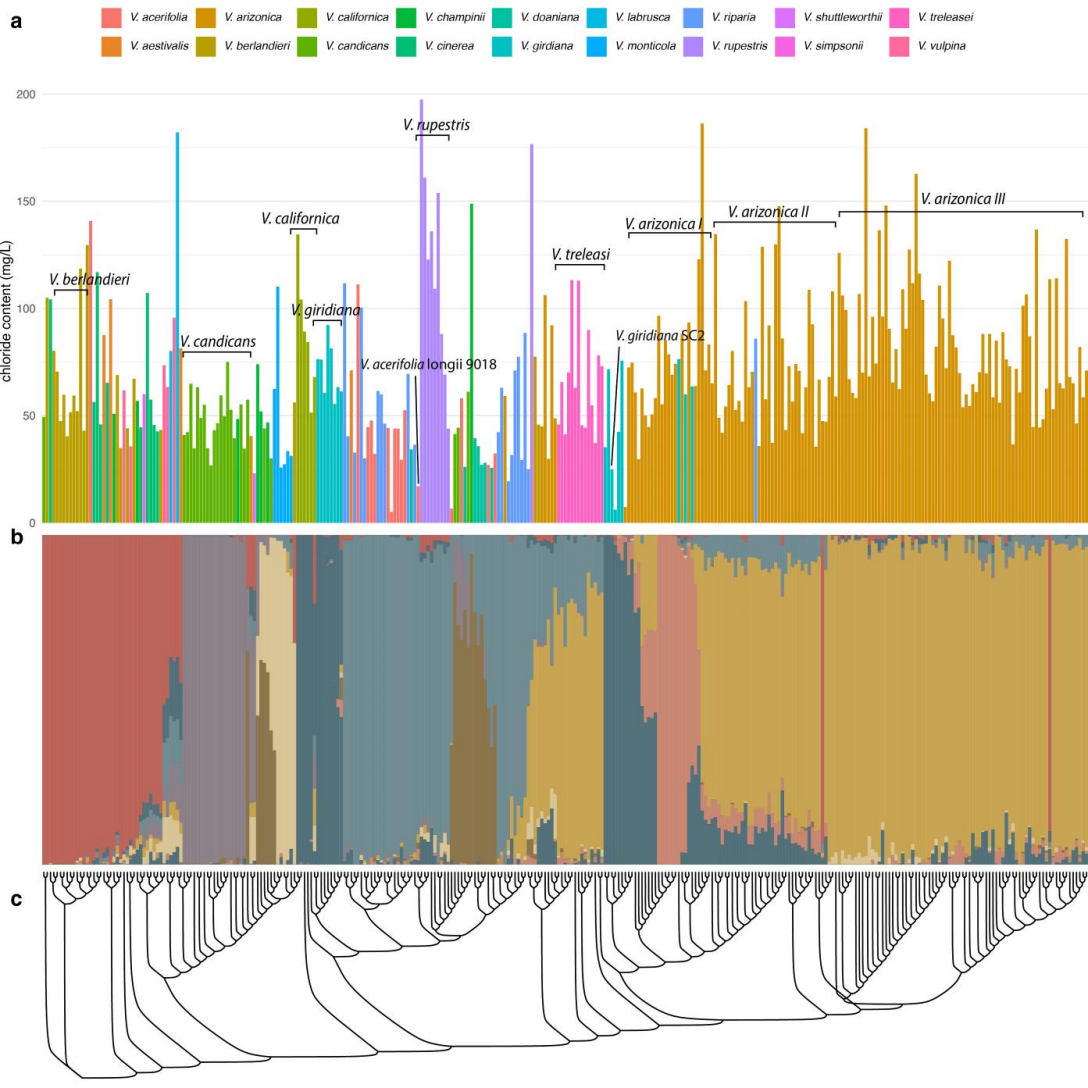
Population structure and phylogenetic relationships among 313 Vitis accessions with varying chloride exclusion. a) Chloride content in each accession, arranged in the same order as the phylogeny in (c). Bars are colored according to species. b) Structure analysis showing ancestry proportions for each accession (vertical bars), with colors representing different genetic clusters (unrelated to the species colors in a). Accessions are arranged as in (c). c) Neighbor-joining phylogenetic tree.

We combined whole-genome sequence data from 2 previous studies, which included 313 of the accessions used in our chloride screening (Morales-Cruz *et al.* 2021; Cochetel *et al.* 2023; Morales-Cruz *et al.* 2023). We performed a new SNP-calling analysis using the haplotype 1 genome of *V. girdiana* SC2 (Cochetel *et al.* 2023), yielding 2,336,705 markers (an average of 122,984 per chromosome) with a MAF above 0.1. We examined population structure and phylogeny to infer the demographic history of *Vitis* and its impact on chloride exclusion (Fig. 2a). Genetic structure analysis revealed 9 distinct ancestral genetic pools, which were differentially present among the individuals examined (Fig. 2b). Based on this, several species, such as *V. berlandieri*, *V. candicans*, *V. californica*, and *V. rupestris*, formed well-defined groups with little to no admixture. Interestingly, even within these uniform groups, chloride exclusion varied by >4-fold for some species (e.g. *V. rupestris* and *V. berlandieri*), likely due to adaptive introgressions through hybridization or genetic differentiation driven by local adaptation. Other species, such as *V. vulpina*, *V. labrusca*, and *V. treleasei*, exhibited more mixed ancestry. The neighbor-joining tree (Fig. 2c) further illustrated these relationships.

**Fig. 3.**
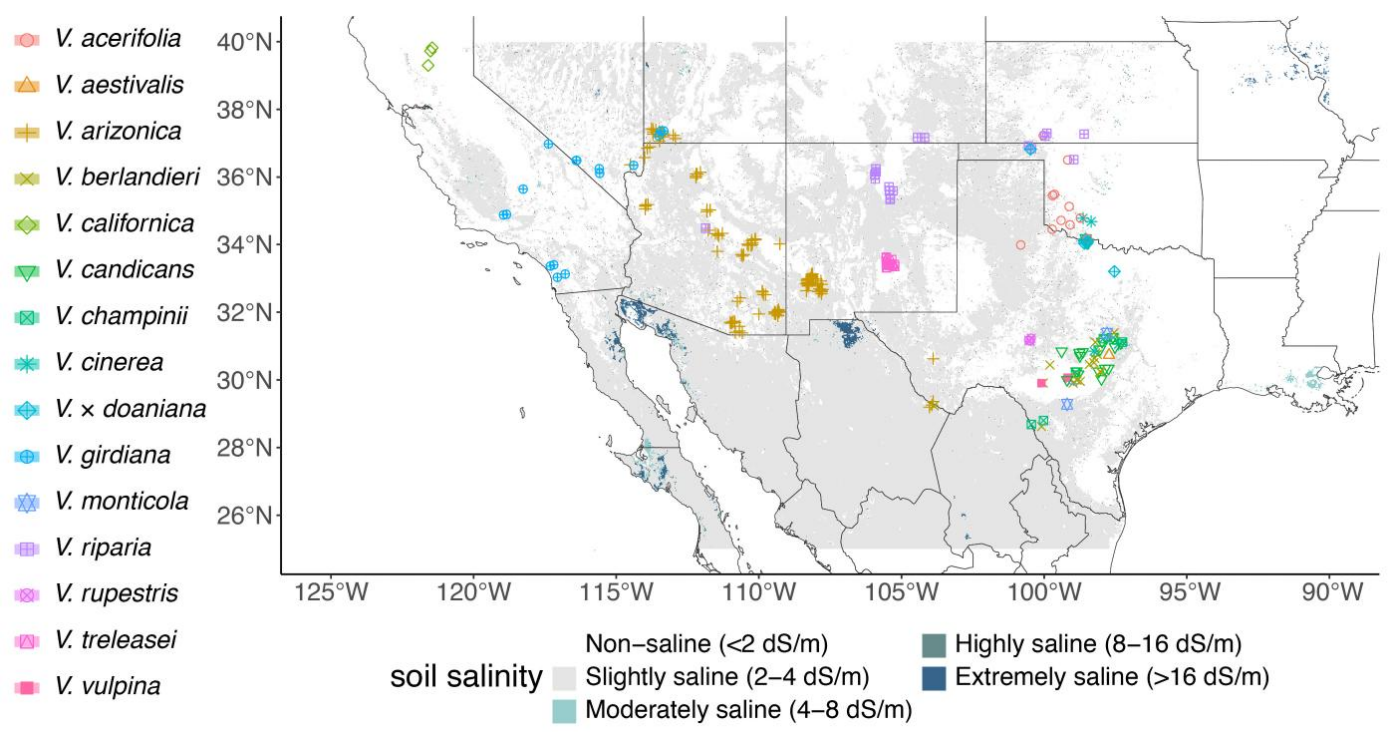
Geographic distribution, soil salinity, and chloride-exclusion variation among 256 *Vitis* accessions. Spatial distribution of accessions across the southwestern United States and northern Mexico. Background shading represents soil salinity levels, classified as nonsaline (<2 dS/m), slightly saline (2 to 4 dS/m), moderately saline (4 to 8 dS/m), highly saline (8 to 16 dS/m), and extremely saline (>16 dS/m), based on data from the WoSIS database.

To assess whether soil salinity at the original collection sites influences the chloride exclusion capacity of the 256 accessions with available geographic coordinates, we compiled soil salinity data from the global soil salinity map (Ivushkin *et al.* 2019). This dataset was generated using a random forest classifier trained on soil property maps, thermal infrared imagery, and ECe point data from the WoSIS database (Batjes *et al.* 2017). Rather than directly mapping Na⁺ or Cl⁻ concentrations, this dataset relies on ECe as an indicator of total dissolved salts, including sodium, chloride, and other ions. Soil salinity is classified into discrete categories: nonsaline (<2 dS/m), slightly saline (2 to 4 dS/m), moderately saline (4 to 8 dS/m), highly saline (8 to 16 dS/m), and extremely saline (>16 dS/m). Among the *Vitis* accessions with available geographic sampling data, 114 were collected from sites classified as slightly saline (2 to 4 dS/m), while 142 were from nonsaline locations (<2 dS/m; Fig. 3). We found no significant differences between leaf Cl^−^ content and soil salinity class (*F* = 0.9513, *P* = 0.3303). Several explanations can be made, including that this dataset does not truly capture the salinity conditions at the sampling locations of the accessions in this study or that there are other forces driving chloride exclusion, such as climate. We then tested associations between climatic variables compiled with the *EnvRtype* package (Costa-Neto *et al.* 2021) and chloride-exclusion levels. Specifically, the mean temperature of the driest quarter (*F* = 24.76, *P* = 1.21e−06), the max temperature of the warmest month (*F* = 11.79, *P* = 0.000696), and the mean temperature of the warmest quarter (F = 14.64, *P* = 0.000164) were all strongly associated with chloride exclusion, suggesting that heat stress during drier periods may play a role in chloride adaptation. Similarly, precipitation patterns were also relevant, with precipitation of the coldest quarter (*F* = 11.44, *P* = 0.000835), precipitation of the driest quarter (*F* = 11.15, *P* = 0.000965), precipitation seasonality (*F* = 11.51, *P* = 0.000802), and precipitation of the driest month (*F* = 14.08, *P* = 0.000218) all showing significant associations with chloride-exclusion levels (data not shown).

### GWAS revealed loci and candidate genes associated with chloride exclusion

**Fig. 4.**
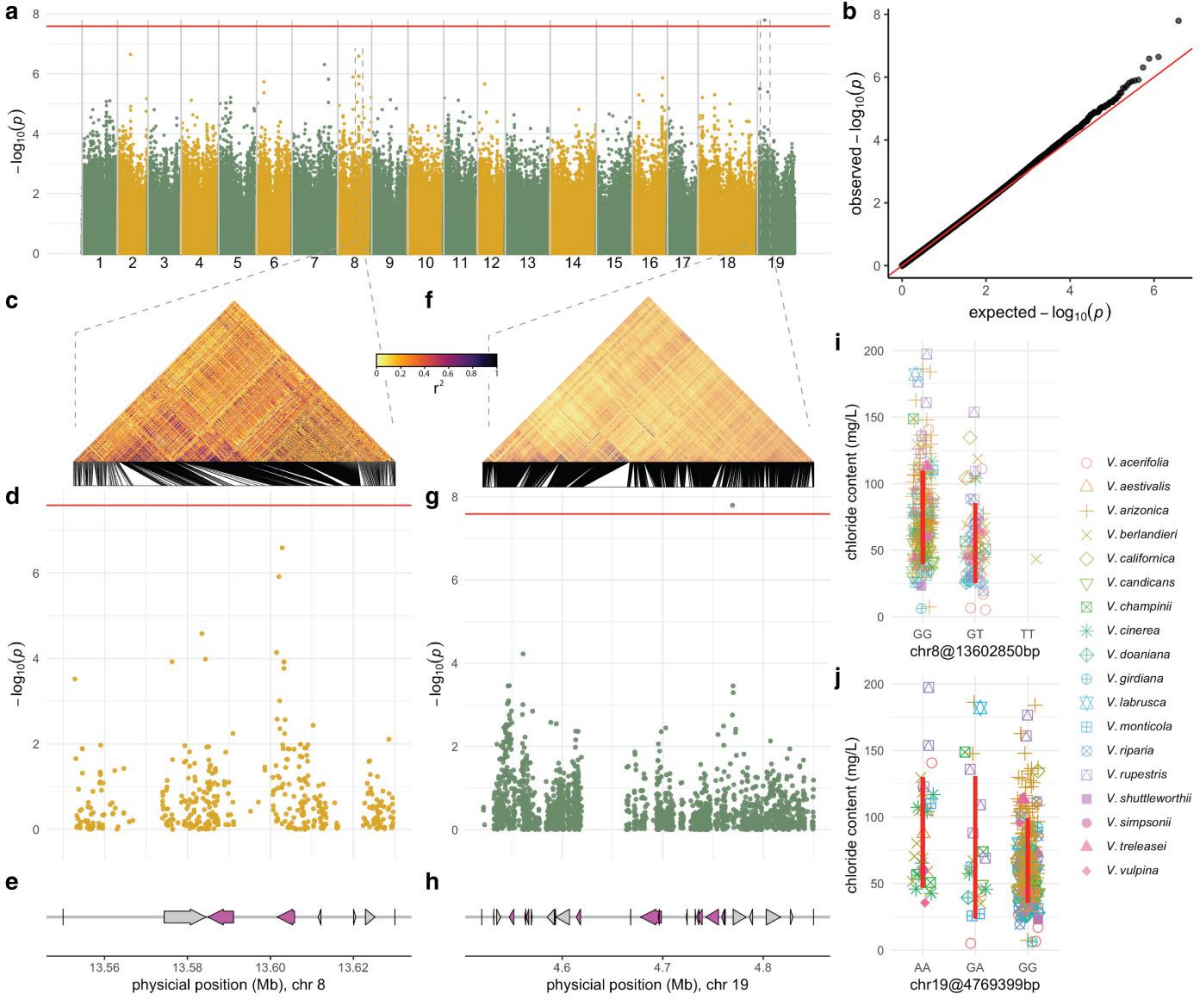
Genome-wide association of chloride exclusion in grapevine. a) Manhattan plot displaying the genome-wide distribution of SNP associations (1,916,527 markers) with chloride exclusion across the grapevine genome *V. girdiana.* The horizontal red line denotes the significance threshold (FDR-adjusted *P*-value 0.05), highlighting 1 significant QTL. b) Quantile–quantile plot assessing the observed vs expected −log”₀(*p*) values under the null hypothesis, indicating deviations due to associations. c) LD heat map (*r*^2^) in the significant region of chromosome 8. d) Local Manhattan plot for chromosome 8, zooming in on the significant region identified in (a). e) Gene content in the significant chromosome 8 region, with genes represented as arrows. Genes of interest (*Cation/H⁺ Exchanger 3*) are highlighted in purple. f) LD heat map (*r*^2^) for the significant region on chromosome 19, analogous to (c). g) Local Manhattan plot for chromosome 19, highlighting significant associations. h) Gene content in the significant chromosome 19 region, similar to (e), where genes are represented as arrows. Multiple copies of the G-type lectin S-receptor-like serine/threonine-protein kinase gene are highlighted in purple. (i, j) Effect plots displaying chloride content (mg/L) across different genotypes for the SNPs at chromosome 8, position 13.6 Mb and chromosome 19, position 4.79 Mb. Vertical red lines correspond to 2 SDs center in the mean by genotype.

GWAS identified a single variant on chromosome 19 of *V. girdiana* haplotype 1 genome (position 4,769,399 bp) significantly associated with chloride exclusion (beta = 24.75 mg/L; Fig. 4a), representing a novel QTL. At a more relaxed threshold −log_10_(*p*) = 6 (instead of −log_10_(*p*) = 7.58, corresponding to the FDR-adjusted threshold), we detected 3 additional associations on chromosomes 2 (8,523,304 bp, beta = 22.72 mg/L), 7 (21,049,011, beta = −18.89 mg/L), and 8 (13,602,850, beta = −29.78 mg/L). The chromosome 2 and 7 loci have not been previously reported, whereas the chromosome 8 locus coincides with a known QTL at the same region (13,598,495 bp; Cochetel *et al.* 2023). This overlap is likely because nearly half of the accessions used in our study, which span 12 *Vitis* species, were also included in the study by Cochetel *et al.* (2023), which reused phenotypic data originally generated by Heinitz *et al.* (2020) as part of a large salinity trial assessing chloride exclusion. However, all phenotypic data in this study, including for overlapping accessions, were newly generated and not reused from prior studies.

To account for population structure, we incorporated both a kinship matrix (from the full marker set) and 10 principal components (PCs), which explained ∼85% of the variation. A Q–Q plot confirmed that these corrections mitigated population stratification (Fig. 4b). The full GWAS results, including *P*-values and effect sizes for all markers, are provided in Supplementary File 1.

Examination of the chromosome 8 QTL revealed a cluster of linked SNPs with rapid LD decay (Fig. 4c), especially among those with the highest −log_10_(*p*) values (Fig. 4d). As reported by Cochetel *et al.* (2023), this region contains 2 genes (*g144880*, 13,584,884 to 13,591,103 and *g144890*, 13,601,610 to 13,605,816) encoding predicted homologs of the Arabidopsis cation/H^+^ exchanger *AtCHX20* (*AT3G53720*; Fig. 4e). The GWAS results for chromosome 8 using unfiltered marker set are provided in Supplementary File 2. A similar LD pattern was observed around the novel QTL on chromosome 19 at 4,769,399 bp (Fig. 4f). The upstream region (Fig. 4g) contains 5 copies (*g307480* at 4,562,881 to 4,563,707; *g307550* at 4,667,975 to 4,668,211; *g307570* at 4,695,533 to 4,696,547; *g307590* at 4,732,332 to 4,732,826; *g307640* at 4,761,909 to 4,762,151) of a gene predicted to encode a G-type lectin S-receptor-like serine/threonine-protein kinase homologous to *At4g27290* (Fig. 4h). The GWAS results for chromosome 19 using unfiltered marker set are provided in Supplementary File 3. Four additional genes annotated as G-type lectin S-receptor-like kinases (RLKs; *g307500*, *g307610*, *g307620*, *g307630*) were also identified in this region. This region is promising, as G-type lectin S-receptor-like serine/threonine-protein kinases have been identified as positive regulators of salt stress tolerance in multiple crops (Joshi *et al.* 2010; Sun *et al.* 2013, 2018). A full list of genes in the QTL intervals of chromosomes 2, 7, 8, and 19 is provided in Supplementary Table 4.

### QTL mapping identifies 2 CHX genes

While GWAS is a powerful tool for detecting associations between genetic variants and traits in diverse, often unrelated populations, QTL mapping serves a complementary role by leveraging the controlled genetic structure of a mapping population. In this study, we performed QTL mapping to validate and compare results from GWAS, as well as to identify additional loci that may have gone undetected in the GWAS analysis. QTL mapping also enables the detection of rare alleles segregating within specific families, which may be absent or underpowered in the broader GWAS panel. Together, GWAS and QTL mapping provide a more comprehensive understanding of the genetic architecture underlying chloride exclusion.

To perform QTL mapping, we generated a mapping population derived from the chloride-excluding accession *V. acerifolia* longii 9018 and the commercial rootstock GRN3. Longii 9018 has exceptional chloride-exclusion capabilities (chloride content: 16.97 mg/L, based on the GWAS screening, Fig. 2a). It has previously been reported as a chloride excluder (Heinitz *et al.* 2020; Sharma *et al.* 2024), making it a strong candidate for this study. GRN3 is a nematode-resistant rootstock with moderate salinity tolerance (Andrew Walker, personal communication) and has *V. champinii*, *Vitis rufotomentosa*, and *V. riparia* in its parentage.

**Fig. 5.**
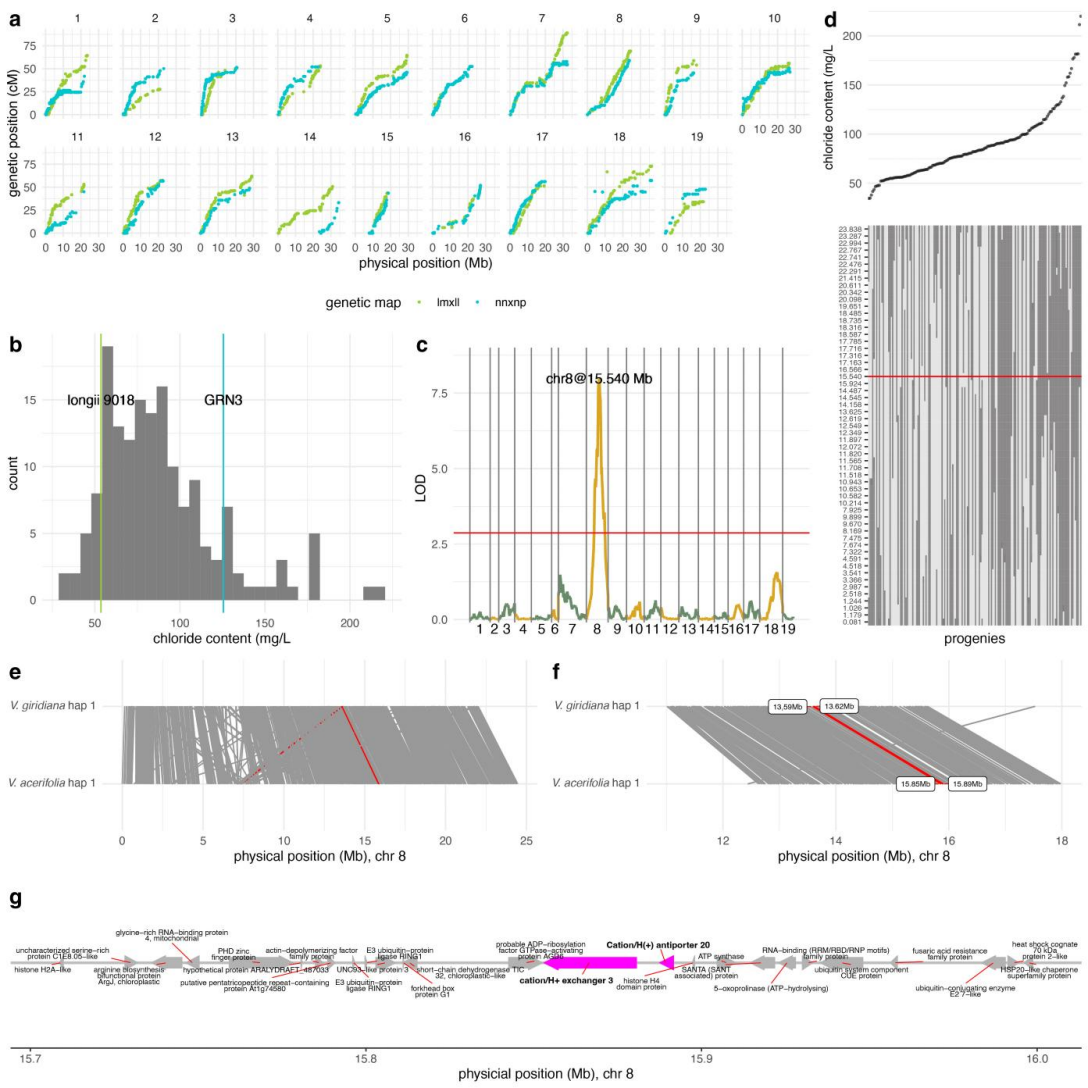
QTL mapping of chloride exclusion in the longii 9018 × GRN3 mapping population. a) Relationship between genetic and physical positions for each chromosome in the parental maps (lm × ll in green, nn × np in blue). b) Distribution of chloride content in the mapping population, estimated using BLUPs. The parental values are indicated with vertical lines: longii 9018 (green) and GRN3 (blue). c) QTL scan for chloride exclusion based on the parental lm × ll map. The LOD scores along the genome are shown, with a peak detected on chromosome 8 at 15.540 Mb (chr8@15.540 Mb). The red horizontal line represents the significance threshold obtained from 1,000 permutations at the 95th percentile. d) Segregation analysis of chromosome 8 genetic markers and their relationship with chloride exclusion. Progenies (columns) are sorted by increasing chloride content (top panel). The genotype matrix (bottom panel) represents genotypic segregation across chromosome 8, with dark gray indicating homozygous (ll) and light gray indicating heterozygous (lm) individuals. The red horizontal line corresponds to the major QTL peak from (c). e) Synteny along chromosome 8 between *V. giridiana* (haplotype 1) and *V. acerifolia* (haplotype 1). f) Zoomed-in view of the syntenic region identified through GWAS, which contains the 2 *cation/H^+^ exchanger 3* genes. g) Gene content based on the *V. acerifolia* longii 9018 genome (haplotype 1; used for SNP calling) across the candidate region (15.45 to 15.65 Mb).

The longii 9018 × GRN3 mapping population (*n* = 162), referred to here as 18113, was genotyped using GBS, with variant calling based on the genome of *V. acerifolia* longii 9018 (Cochetel *et al.* 2023). Two genetic maps were constructed using a pseudo-test cross linkage mapping strategy (Grattapaglia and Sederoff 1994). The parental map for longii 9018, which contained polymorphic markers (ABxAA or lmxll), included 2,611 markers across 740 bins (unique genetic positions). The parental map for GRN3 contained 4,842 markers across 784 bins. Physical and genetic distances showed strong agreement for both parental maps (Fig. 5a).

The mapping population was screened for chloride exclusion using the same method as above (Heinitz *et al.* 2020; Sharma *et al.* 2024). The screening was conducted in a semi-augmented design across 4 trials, with varying numbers of genotypes per trial (*n* = 21, 21, 55, and 143). Among the progeny, 87 were included in only 1 trial, while 73 were tested in 2 trials. Longii 9018 was included in all trials, whereas GRN3 was included in 3. Transgressive segregation was observed, with chloride content ranging from 34.46 to 219.96 mg/L and a mean of 88.69 mg/L (Fig. 5b). The parental genotypes, longii 9018 and GRN3, exhibited chloride contents of 53.62 and 125.61 mg/L, respectively. These values were lower than those observed when the same accessions were evaluated in the diversity panel, although their relative ranking remained unchanged. This discrepancy is not unexpected, as variation across trials is common and highlights the importance of including control accessions to enable meaningful comparisons. We used the scanone function from the R package qtl (Broman *et al.* 2003) to identify QTLs in each parental map separately. A major QTL was detected on chromosome 8 at 41.16 cM, with a logarithm of odds (LOD) score of 8.01, explaining 20.6% of the variance (Fig. 5c). This QTL was found on the parental map with markers segregating for longi 9018 (ABxAA or lmxll). Further examination of the segregants and their phenotypes at the QTL showed lower chloride content in heterozygous genotypes (Fig. 5d, lighter gray) and higher chloride content in homozygotes (darker gray). The QTL marker was located at 15,540,293 bp in the longii 9018 haplotype 1 genome. Just downstream (within the 1.5-LOD support interval), between 15,853,017 and 15,891,666 bp, 2 contiguous genes were identified with predicted annotations: *Cation/H^+^ Exchanger 3* (*g154540*: 15,853,017 to 15,880,628) and *Cation/H(+) Antiporter 20* (*g154550*: 15,887,484 to 15,891,666). Using minimap2 (Li, 2018), we found that this region (chromosome 8, 15,853,017 to 15,891,666) is orthologous to the *V. girdiana* region in 13.60 Mb containing the 2 *Cation/H^+^ exchangers* identified through GWAS (Fig. 5, e, f, g). No QTLs, or any sign of significance, were found using GRN3 parental map, which indicates that this chloride-exclusion locus is inherited from the *V. acerifolia* longii 9018 parent.

### Genetic variants located in the *cation/H⁺ AtCHX20 exchanger* gene

**Fig. 6.**
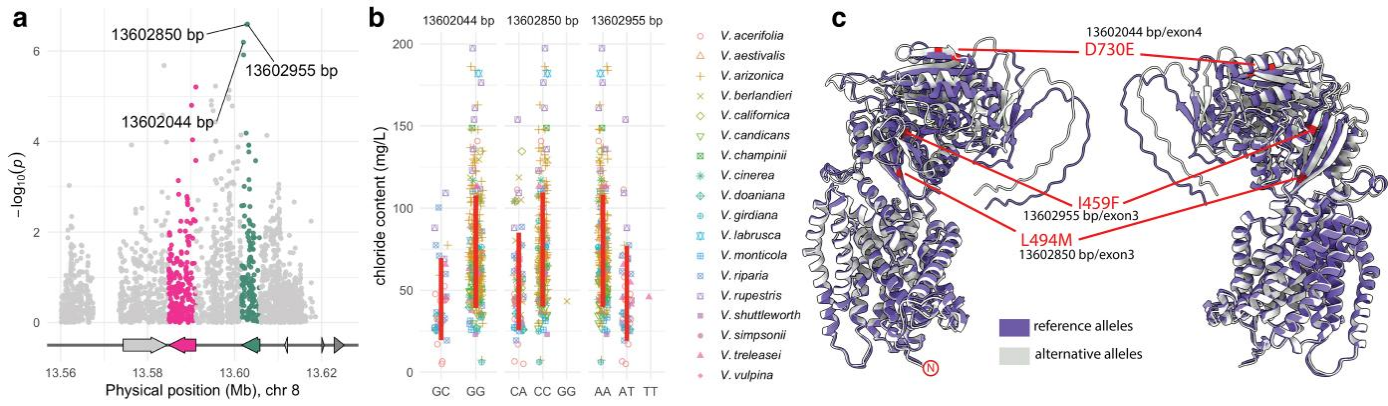
Identification of exonic variants in the cation/H⁺ *AtCHX20* exchanger gene associated with chloride exclusion. a) Manhattan plot displaying the association of SNPs on chromosome 8 (13.56 to 13.62 Mb) with chloride content. The most significant markers are located in the second copy (*g144890*, green arrow) of a predicted CHX homolog, with 3 SNPs (13,602,044, 13,602,850, and 13,602,955 bp) showing high −log”₀(*p*) values. The *g144880* gene and markers within it are colored in pink. b) Effect plots displaying chloride content (mg/L) across different genotypes for 3 nonsynonymous changes at chromosome 8 for SNPs 13,602,044, 13,602,850, and 13,602,955. Vertical red lines correspond to 2 SDs center in the mean by genotype. c) Structural model of the predicted CHX protein, comparing the reference allele conformation (purple) with the alternative allele conformation (gray). The 3 nonsynonymous mutations (D730E, L494 M, and I459F) are highlighted in red, indicating their positions within the exon 3 and exon 4 regions of *g144890*.

Considering the agreement between GWAS and QTL mapping, we further inspected the QTL region 13.56 to 13.62 Mb on chromosome 8 using an unfiltered (MAF > 0.05) version of the VCF for this region. Using GWAS, 5,216 markers were tested for association with chloride content. Kinship and 10 PCs derived from the genome-wide marker dataset were included in the GWAS model. The most significant markers were found in the second copy (*g144890*, 13,601,610 to 13,605,816) of the predicted homologs of the Arabidopsis CHX *AtCHX20* (Fig. 6a). Four SNPs (13,602,044; 13,602,071; 13,602,850; 13,602,955) within this gene had significantly higher −log_10_(*p*) values (>5.5) compared with other surrounding markers. Minor allele frequencies for these SNPs ranged from 0.062 to 1.131. The *g144890* gene contains 4 exons, with the first 2 associated with the Sodium/Hydrogen Exchanger Transmembrane Domain (Pfam entry: PF00999) and the last 2 with the Plant Cation/H⁺ Antiporter Domain (Pfam entry: PF23256). All 4 SNPs were in exons 3 and 4. Among these SNPs, 3 resulted in nonsynonymous amino acid changes: D to E (13,602,044, G/C), M to L (13,602,850, C/A), and I to F (13,602,955, A/T). The 13,602,071 (G/A, R/R) SNP resulted in a synonymous mutation with no amino acid change. Marker effects (Fig. 6b) for the 3 nonsynonymous changes were equal to or more pronounced than those observed in the GWAS with the genome-wide marker set (Fig. 4i).

To further investigate the structural and functional consequences of these nonsynonymous substitutions, we used AlphaFold (Jumper *et al.* 2021) to predict the protein structures of the reference allele (wild type for all 3 SNP positions) and the alternative allele (mutant for all 3 SNP positions). Visually, no major structural alterations were observed between the 2 predicted protein conformations (Fig. 6c).

Although the overall structure remained unchanged, individual amino acid substitutions may still influence local interactions. The aspartate (D) to glutamate (E) change is conservative, as both residues are negatively charged. However, glutamate's longer side chain may introduce minor steric effects that could influence interactions, particularly in tight or functionally critical regions. The methionine (M) to leucine (L) substitution is also conservative due to their shared hydrophobic nature, but the loss of methionine's sulfur-containing side chain could affect redox sensitivity or metal-binding properties if relevant to the protein's function. In contrast, the phenylalanine (F) to isoleucine (I) change is less conservative, as it removes an aromatic ring, potentially disrupting π-stacking interactions or ligand binding. This alteration could also reduce local stability in regions where aromatic interactions are structurally significant. While these changes appear relatively mild, their potential functional consequences and role in differential chloride affinity require further investigation.

The 4,560,000 to 4,900,000 bp region of chromosome 19 was also inspected using 21,789 unfiltered SNP variants. However, the most significant marker was in an intergenic region (Supplementary Fig. 1), and no further candidate gene search analyses were pursued.

## Discussion

### The diversity in chloride exclusion and the role of hybridization and local adaptation

The extensive phenotypic variation in leaf chloride content across *Vitis* species underscores the complexity of chloride exclusion, arising from diverse genetic and physiological mechanisms. Additionally, environmental factors such as soil composition, climate variability, and biotic interactions further influence these processes, contributing to the species’ differential responses to salinity stress. Here, we detected a broader range of chloride exclusion than previously reported. Our findings corroborate earlier observations regarding species that excel (*V. acerifolia*, *V. × doaniana*) and those that perform poorly (*V. rupestris*, *V. labrusca*; Gong *et al.* 2011; Fort *et al.* 2013; Heinitz *et al.* 2020; Sharma *et al.* 2024). Recognizing the large intraspecific diversity is crucial, especially since traits like salinity tolerance are often viewed as fixed, species-level attributes (Rahemi *et al.* 2022).

Interspecific compatibility in *Vitis*, coupled with overlapping habitats, actively promotes hybridization—a key driver of phenotypic and structural genomic variability (Callen *et al.* 2016; Morales-Cruz *et al.* 2021; Cantu *et al.* 2024). In southwestern North America, multiple wild *Vitis* species frequently co-occur in canyon or riparian corridors, facilitating spontaneous introgression and admixture. Recent analyses of haplotype-resolved genome assemblies and a pangenome have revealed pervasive hemizygosity and structural variation—such as large inversions, translocations, and gene copy-number changes—across diverse *Vitis* lineages (Cochetel *et al.* 2023). This structural variation can directly impact essential phenotypic traits, including responses to soil salinity and other adaptation traits. These admixture patterns raise important questions about the degree to which physical barriers (e.g. desert basins, mountains) and local edaphic or climatic factors limit gene flow among overlapping wild populations. It also underscores cases wherein regionally confined species (e.g. *V. treleasei*) still display phenotypic breadth on par with widely distributed taxa like *V. arizonica*. Determining whether local gene flow among co-occurring species outweighs soil- or climate-driven selection requires further investigation.

We note that our inferences about *Vitis* chloride-exclusion phenotypes are shaped by the study's genetic and geographic sampling, which could introduce bias—particularly for under-sampled species. Additionally, given the interspecific compatibility of *Viti*s species and their ability to produce fertile hybrids, some degree of species misclassification is possible.

### On the use of trans-species GWAS

Our study implemented a multi-species (trans-species) GWAS by pooling individuals from 18 *Vitis* species to map genetic loci linked to chloride exclusion. This approach departs from the traditional single-species GWAS and raises understandable concerns about population structure and species-specific allele effects. Combining divergent gene pools could introduce confounding if not properly controlled. However, in the genus *Vitis*, the boundaries between species are often blurred by gene flow; natural hybridization and introgression are central evolutionary processes in *Vitis* genus (Cantu *et al.* 2024). Because of this extensive interspecific admixture, many alleles underlying adaptive traits, such as chloride exclusion, are shared across species lines. Therefore, a cross-species GWAS can be effective and biologically appropriate in this system, as it leverages those shared genetic variants rather than being misled by strict species differences. There are several advantages to the multi-species GWAS in our case. First, pooling multiple species increases the total genetic and phenotypic diversity examined, which boosts the power to detect QTL that might be missed in a smaller, single-species cohort. The presence of introgressed alleles means that the same beneficial variant may occur in different *Vitis* backgrounds (Fig. 6b); analyzing them together effectively raises the allele frequency and enhances the signal for association. We took care to mitigate potential confounding from interspecific structure by including robust population structure controls in our model (e.g. PCs or kinship matrices capturing species relationships). This ensures that any association we detect is driven by a locus consistently influencing the trait, rather than by systematic differences between species groups. In essence, the multi-species GWAS tests for genotype–phenotype links that transcend any 1 species—an approach that is valid here because gene flow has made the Vitis gene pool a continuum rather than discrete units.

### Two independent experiments point out *CHX3* as a candidate gene for chloride exclusion in grapevines

Salinity tolerance in grapevines involves multiple genetic loci, regulating sodium and chloride uptake, transport, and tissue partitioning (Wu *et al.* 2019). Historically, research has focused more on sodium exclusion, given its critical role in major crops (Munns and Tester 2008; Henderson *et al.* 2018). However, grapevines are often more susceptible to chloride toxicity, which manifests as marginal leaf burn, reduced photosynthesis, compromised fruit quality, and overall vine decline (Walker 1994; Tregeagle *et al.* 2010). Consequently, the ability to exclude or compartmentalize chloride from shoot tissues is widely recognized as a key determinant of salinity tolerance in *Vitis* (Antcliff *et al.* 1983; Sykes 1985; Fort *et al.* 2015; Heinitz *et al.* 2020). Despite significant progress in identifying sodium-exclusion mechanisms—most notably *VvHKT1;1*, which functions in retrieving sodium from xylem vessels into pericycle cells (Henderson *et al.* 2018)—the genetic basis of chloride exclusion has remained largely elusive. Several hypotheses have been proposed, including SLAH1 to SLAH3–mediated chloride efflux at the root pericycle (Cubero-Font *et al.* 2016), direct chloride extrusion from roots (Abbaspour *et al.* 2013; Wu *et al.* 2019), and shoot-to-root recirculation of chloride (Godfrey *et al.* 2019; Qu *et al.* 2021). The relative complexity of these processes may explain why both single-locus (Antcliff *et al.* 1983) and polygenic (Sykes 1985; Fort *et al.* 2015) models have been suggested for chloride exclusion in grapevines.

Recently, Cochetel *et al.* (2023) identified a QTL associated with chloride exclusion on chromosome 8, which colocalized with 2 genes encoding proteins homologous to the *A. thaliana* CHX AtCHX20 (*AT3G53720*). Our study, using 2 independent mapping approaches, GWAS and QTL mapping, confirmed this locus with a dataset nearly twice the size of that used by Cochetel *et al.* (2023) and employing 2 different genomes for variant calling (*V. girdiana* and *V. acerifolia*). AtCHX20 is a member of the cation/proton antiporter (CPA2) family in Arabidopsis, primarily involved in ion homeostasis and pH regulation within plant cells. It plays roles in guard cell movement, osmoregulation, and membrane trafficking (Padmanaban *et al.* 2007; Chanroj *et al.* 2012). Phylogenetic analyses suggest that *AtCHX20* and its homologs originated from cyanobacterial NhaS4-like genes and diversified in early land plants, leading to specialized functions in ion transport and cellular adaptation to environmental stress (Sze *et al.* 2004). In Arabidopsis, *AtCHX20* localizes to endomembrane, where it influences vesicular trafficking and intracellular pH balance—key processes that aid in plant adaptation to fluctuating environmental conditions. Other family members, such as *AtCAX3* and *AtCAX4*, have been shown to be upregulated under salt stress (Cheng *et al.* 2002), while the overexpression of rice CAX (*OsCHX11*) improved salt tolerance (Senadheera *et al.* 2009). In soybean, *GmCHX1* was also identified as a major gene conferring salt tolerance (Do *et al.* 2019).

The discovery that *Vitis* homologs of *AtCHX20* colocalize with a QTL for chloride exclusion suggests a potential role of cation/proton exchangers in chloride homeostasis, either through compartmentalization or by modulating ion fluxes that indirectly affect chloride transport. However, based on findings from Heinitz *et al.* (2020) and Cochetel *et al.* (2023), it is evident that this locus on chromosome 8 does not regulate chloride root compartmentalization. Chloride concentrations in root tissues showed no correlation with either leaf chloride concentrations or the genotype at this locus, reinforcing the idea that the mechanism of exclusion linked to this locus does not involve root sequestration.

While we identified nonsynonymous substitutions within CHX gene in the QTL region on chromosome 8, we acknowledge that the evidence for these variants being causative remains circumstantial and is not yet supported by functional validation. It is possible that regulatory or structural variants in this region, rather than coding mutations, underlie the observed phenotypic differences in chloride exclusion. Further studies integrating transcriptomic or pangenomic approaches are needed to clarify the mechanisms involved and to fully resolve the genetic basis of this locus. However, the identification of what appears to be a large-effect QTL for chloride exclusion on chromosome 8 represents a big opportunity in rootstock breeding, as it provides an avenue for rapid allele introgression through marker-assisted selection (MAS). Historically, grape breeding has prioritized single-gene traits primarily in scion breeding, focusing on traits such as resistance to powdery mildew (Coleman *et al.* 2009; Pap *et al.* 2016), downy mildew (Schwander *et al.* 2012; Bhattarai *et al.* 2021; Sapkota *et al.* 2023), and Pierce's disease (Krivanek *et al.* 2006; Riaz *et al.* 2006), as well as improvements in fruit quality (Chen *et al.* 2015; Wang *et al.* 2020; Reshef *et al.* 2022). Many of these traits have been introgressed from wild *Vitis* species into *V. vinifera* backgrounds (Svyantek *et al.* 2020; Thomas *et al.* 2020; Morales-Cruz *et al.* 2023). In contrast, candidate gene discovery for rootstock traits has been far more limited, with only a few notable exceptions, such as *XiR1*, which confers resistance to dagger nematode (Xu *et al.* 2008). The discovery of a genetic locus associated with chloride exclusion provides a critical tool for developing rootstocks with enhanced salinity tolerance, surpassing what is currently achievable with commercially available options like 140Ru. The ability to introgress this trait efficiently into elite rootstock germplasm through MAS could reduce the time and cost required to develop new, more resilient varieties. Additionally, understanding the functional mechanism of CHX-type transporters in chloride exclusion could inform transgenic or gene-editing approaches to further optimize rootstock performance under saline conditions.

Given its relevance for breeding, we have designated the locus on chromosome 8 associated with chloride exclusion as qClEx8.1. This naming reflects its potential utility as a major QTL for developing chloride-tolerant grapevine rootstocks.

### Novel QTLs for chloride exclusion

While other weak and strong associations were identified in our GWAS, the QTL on chromosome 19 stands out as particularly promising. This region contains 9 gene copies predicted to encode G-type lectin S-receptor-like serine/threonine-protein kinases, which have been previously documented as key regulators of ion homeostasis and stress signaling in *Vitis* (Han and Li 2024). These kinases play crucial roles in plant responses to environmental stressors, including salinity, by mediating signal transduction pathways that regulate ion transport, oxidative stress responses, and osmotic balance. Previous studies provide strong evidence for the involvement of RLKs in salt stress responses. For instance, the Ca^2+^-dependent protein kinase VaCPK21 in *Vitis amurensis* is significantly upregulated under salt stress, suggesting a role in stress adaptation (Dubrovina *et al.* 2016). More specifically, G-type lectin S-RLKs have been identified as positive regulators of salt tolerance across multiple species, including Arabidopsis (Sun *et al.* 2013), pea (Joshi *et al.* 2010), and alfalfa (Sun *et al.* 2018). In alfalfa, overexpression of *GsSRK-f* and *GsSRK-t* enhanced salt tolerance by modulating ion homeostasis, ROS scavenging, and osmotic regulation (Sun *et al.* 2018). Similarly, overexpression of *PsLecRLK* in tobacco improved Na^+^ compartmentalization and ROS detoxification, thereby increasing salinity tolerance (Vaid *et al.* 2015). In Arabidopsis, *AtLecRK2* and *AtLecRK-b2* are upregulated in response to salt and osmotic stress (He *et al.* 2004; Deng *et al.* 2009), while in rice, G-LecRLKs show increased expression under cold, drought, and salt stress (Vaid *et al.* 2012).

The identification of G-LecRLK in our study suggests that this gene family may contribute to grapevine adaptation to salt stress by modulating stress-responsive signaling cascades. Plants employ a combination of strategies to cope with salinity, including ion exclusion, osmotic regulation, and oxidative stress management (Xiong *et al.* 2002). The genes identified in this study—*CHX3* and *G-LecRLK*—may function in concert to enhance chloride exclusion and overall salt tolerance in grapevines. Alternatively, they may represent distinct adaptation mechanisms that vary across species or even among individual accessions, highlighting the complexity of salinity tolerance in *Vitis*. Further functional validation of these loci will be essential to unravel their precise roles in grapevine salt stress responses.

Future research should focus on validating the functional role of CHX homologs and *G-LecRLK* in *Vitis*, possibly through transcriptomic, physiological, and CRISPR-Cas9 knockout studies. Additionally, further work is needed to investigate the potential interplay between this chloride-exclusion mechanism and other abiotic stress responses, including drought and nutrient uptake efficiency. Given that chloride-exclusion mechanisms in *Vitis* remain underexplored, the findings of this study set the stage for further advances in both fundamental and applied plant breeding research.

## Supplementary Material

jkaf149_Supplementary_Data

## Data Availability

All the collected data have been made available in the Supplementary Tables 1 to 4 accompanying this manuscript, and the Supplementary Files 1 to 3 and GWAS results have been deposited in https://doi.org/10.6084/m9.figshare.28557530. Supplemental material available at G3 online.
