# Correction to: The genetic basis of chloride exclusion in grapevines

**DOI:** 10.1093/g3journal/jkaf287

**Published:** 2026-01-02

This is a correction to: Sadikshya Sharma, Noe Cochetel, Jose R Munoz, Hollywood Banayad, Yaniv Lupo, Veronica Nunez, Ana Gaspar, Christopher Chen, Krishna Bhattarai, Brandon S Gaut, Dario Cantu, Luis Diaz-Garcia, The genetic basis of chloride exclusion in grapevines, *G3 Genes|Genomes|Genetics*, Volume 15, Issue 9, September 2025, jkaf149, https://doi.org/10.1093/g3journal/jkaf149

The following changes have been made to the originally published manuscript.

The statement under **Funding** has been emended to read: “This project was partially funded by the American Vineyard Foundation (project 2023-2781), the California Grape Rootstock Improvement Commission (project A24-0828), the USDA National Institute of Food and Agriculture Specialty Crop Research Initiative (projects 2022-51181-38240 and 2024-51181-43236), and the National Science Foundation grant #1741627.” instead of: “This project was partially funded by the American Vineyard Foundation (project 2023-2781), the California Grape Rootstock Improvement Commission (project A24-0828), the USDA National Institute of Food and Agriculture Specialty Crop Research Initiative (2022-51181-38240), and the National Science Foundation grant #1741627.”

