# Supplementary figures and images for: The genetic basis of chloride exclusion in grapevines
Source: G3 (Bethesda). 2025 Jun 30;15(9):jkaf149. doi: 10.1093/g3journal/jkaf149 (PMC12405893; doi:10.1093/g3journal/jkaf149)

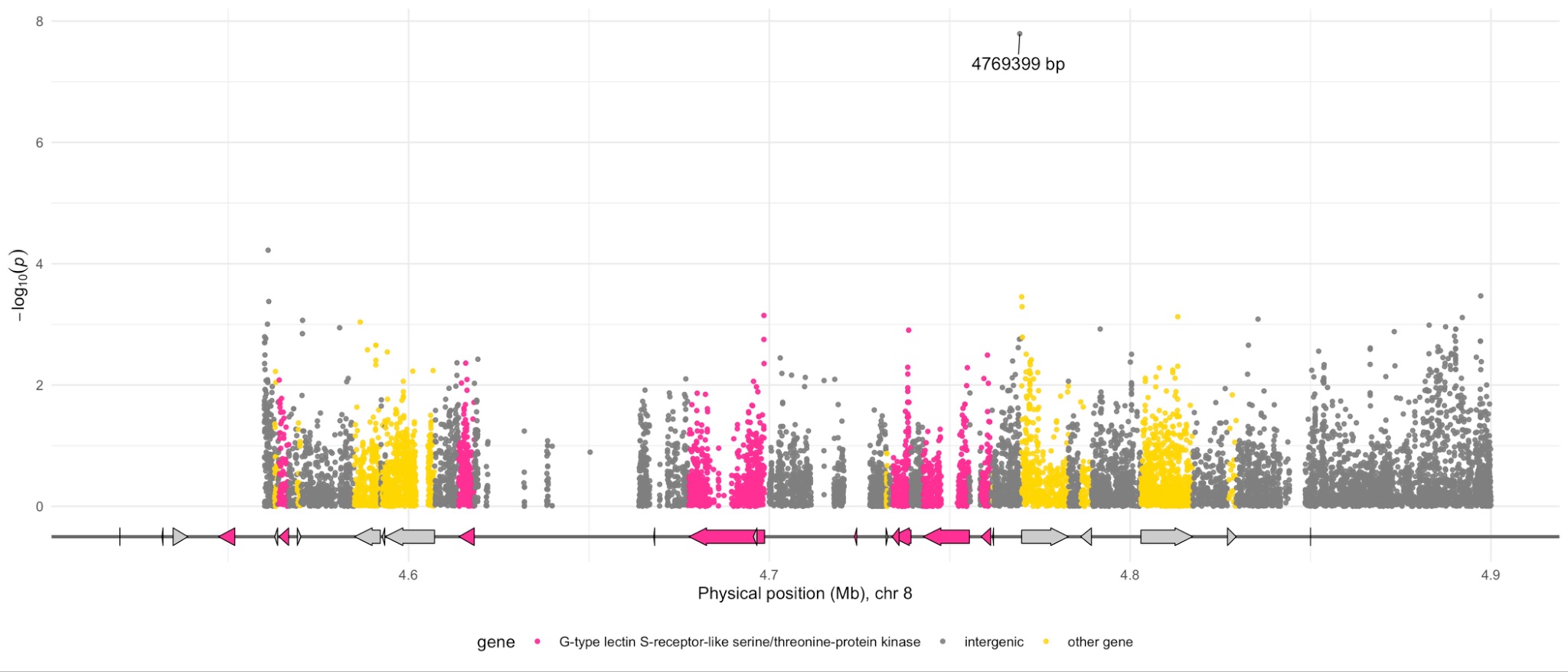


**Supplementary Fig. 1**. Zoomed view of the QTL in chromosome 19 (4.56-4.9 Mb).

Supplement: jkaf149_Supplementary_Data [file jkaf149_supplementary_data.zip › Supplementary_Figure_1_G3-2025-405758.docx]
